# Effects of intestinal parasitic infections on nutritional status of primary children in Imo State Nigeria

**DOI:** 10.11604/pamj.2019.33.34.17099

**Published:** 2019-05-16

**Authors:** Onyenonachi Charity Ihejirika, Obioma Chebechi Nwaorgu, Chikere Ifeanyi Ebirim, Callistus Muodebe Nwokeji

**Affiliations:** 1 Department of Parasitology and Entomology, Nnamdi Azikiwe University Awka, Anambra, Nigeria; 2 Department of Parasitology and Entomology, Nnamdi Azikiwe University Awka, Anambra, Nigeria; 3 Department of Public Health Technology, Federal University of Technology Owerri, Nigeria; 4 Department of Medical Microbiology and Parasitology, Federal Medical Centre, Owerri, Nigeria

**Keywords:** Malnutrition, intensity, prevalence, association, parasitemia, children, intestinal, age, helminth, anthropometric

## Abstract

**Introduction:**

A cross-sectional study was conducted between the months of April to October 2015, to determine the effects of intestinal parasitic infections (IPIs) on nutritional status of school age children in Owerri and Orlu geographical zones, in Imo State, Nigeria.

**Methods:**

Faecal samples were examined using Kato Katz method and formol-ether concentration techniques, while blood samples were examined using cyamethahaemoglobin method. Anthropometric indices were used as indicators of nutritional status, children whose Height-for-Age, Weight-for-Age and Weight-for-Height were <-2 standard deviation (SD) were classified as stunted, wasted, and underweight respectively.

**Results:**

Total prevalence rate of 16.6% was recorded in the study areas with *Ascaris lumbricoides* (4.0%), *Trichuris trichiura* (0.6%), Hookworm (1.0%) *Taenia sp* (0.3%), *Entaomeba histolytica* (5.3%), *Entamoeba coli* (2.7%) and *Giardia lambia* (2.7) Majority (73.4%) of the children had light intensity. Anthropometric study results showed that 79(31.3%) of the children were malnourished. The prevalence of stunting, under-weight and wasting were higher in uninfected (86.1%, 90.0% and 10%) respectively than in infected children (13.9%, 10.0% and 0.0%) respectively, although not significant at p = 0.857, 0.587 and 0.368 respectively. Prevalence of anaemia was 17.4%, anaemia was insignificantly (p = 0.09) higher in infected (21.1%) than in uninfected (16.5%) children. Children that had co-infection recorded higher prevalence (2.2%) of severe anaemia. There was an association (p = 0.002) between anaemia and intensity of helminth infection. Malnutrition was insignificantly (p = 0.319) higher in children with heavy (100.0%) and moderate (75.0%) intensity of helminth infection than children that had light intensity (41.7%) of helminth infection.

**Conclusion:**

When compared with previous study, there were decline in the prevalence of intestinal parasitic infections and anaemia among school age children. Low intensity parasitemia with intestinal parasites had no significant effect on the malnutrition and haemoglobin profile of the children in the study areas. Therefore, improved sanitation and more deworming efforts should be intensified to ensure further decline in prevalence of intestinal parasitic infections.

## Introduction

Intestinal parasitic infections (IPIs) have continued to pose serious medical and public health problem in developing countries, these infections constitute a global health burden causing clinical mortality in 450 million people, especially in children [1, 2]. An estimation by WHO [3] showed that Ascaris lumbricoides, hookworm and Trichuris trichiura infect 1,450 million, 1,300 million and 1,050 million people worldwide, respectively, while intestinal schistosomiasis affects over 200 million people. Outside morbidity and mortality caused by these parasites, infections with intestinal parasites have been associated with stunting, physical weakness and low academic performance of schoolchildren [3]. Intestinal parasitic infections cause decreased intake in the body's nutrient requirement by their interface with absorptive surfaces, physical obstruction of intestinal lumen, production of proteolytic substances and consumption of nutrients intended for body [4, 5]. The impact of intestinal parasitic infections is more on children due to their vulnerability to nutritional deficiencies [1, 6]. The poor people in developing countries experience a cycle where under nutrition and repeated infections lead to excess morbidity that can continue from generation to generation [7].

## Methods

**Figure 1:**
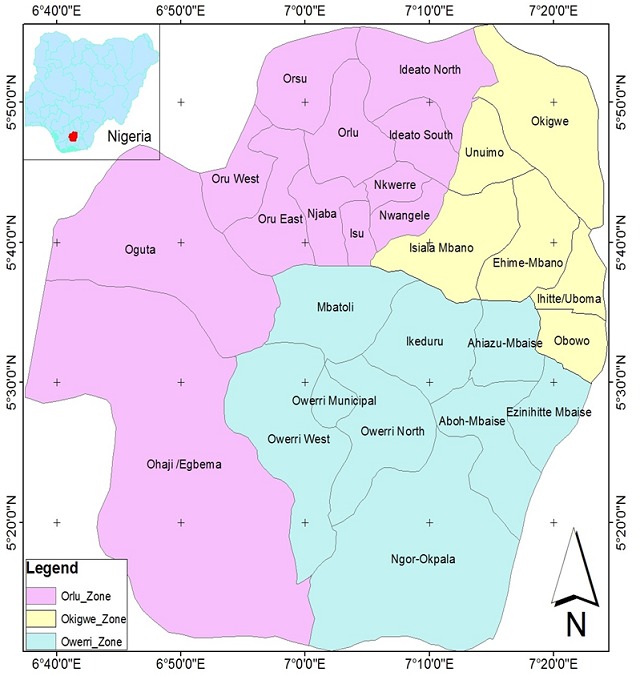
Map of Imo State showing Orlu and Owerri zones

**Study area:** the study was conducted in two zones in Imo State Nigeria in 2015. Imo State is located in South Eastern part of Nigeria. It lies within latitudes 4°45'N and 7°15'N, and longitudes 6°50'E and 7°25'E, with an area of about 5,100 km^2^. The average annual temperature is above 20°C (68.0°F) which creates an annual relative humidity of 75%, with humidity reaching 90% in the rainy season. These areas experience dry season from December to March and Harmattan commences from late December to late January (Figure 1).

**Ethical clearance:** the study was approved by the Department of Parasitology and Entomology, Nnamdi Azikiwe University Awka. Ethical clearance was obtained from Ethical Committee of Imo State Specialist Hospital Umuguma, Owerri Imo State before commencement of the study. Permission to conduct the research was also obtained from the Department of Public Health, Imo State Ministry of Health. Ethical considerations were applied by issuing of results of laboratory analysis to parents. Samples and data from participants were identified with codes and participants were assured of the confidentiality of data collected. The objective of the study was explained to the teachers and parents/guardians of participants, and written informed consent was sought from parents or guardians of selected pupils before commencement.

**Sample and sampling technique:** the sample size was determined using Daniel [8] statistical formula for determination of sample size using prevalence population. One thousand two hundred pupils within the ages of 5-13 years were selected through a random process, (six hundred pupils from each zone) for determination of prevalence of anaemia. Simple random sampling method was also used to select three hundred (300) pupils (150 pupils from each zone) from the already selected 1200 pupils to determine the prevalence of intestinal parasitic infections and anthropometric studies. Pupils with history suggestive of common childhood chronic illness such as sickle cell anaemia, human immunodeficiency virus (HIV) were excluded from the study in accordance with the works of Saloojee and Cooper [9].

**Collection of sample:** every enrolled child was provided with a clean, dry, capped, well-labeled specimen/container bottle for a fresh fecal sample. The pupils were adequately instructed on how to get a little portion of their stool (approximately 5g) into the bottles. Their class teachers assisted in ensuring compliance. At the time of collection, date of sampling, serial number of participant, age, sex and consistency of the stool (formed, soft, semi-soft and watery) were recorded for each subject on a recording format. All stool samples were transported to the Federal Medical Centre Parasitology Laboratory within one hour for analysis. Each faecal sample was examined using Kato Katz method and formol-ether concentration technique. For blood sample, Blood sample (2ml) was collected from each child from the median cubital vein at the elbow using syringe and Tourniqet. The blood was transferred into an EDTA sample bottle for analysis. The blood samples were transported to Federal Medical Centre (Owerri) laboratory within 50 minutes for analysis.

**Anthropometric measurement:** anthropometric measurements of the pupils were carried out by a method described by WHO [10]. The subjects were weighed barefooted and in light clothing on a bathroom scale accuracy of 0.1kg. The scale was standardized before use with 11kg weight. Height was measured to the nearest 1cm, with a paper stadiometer attached to a vertical wall. Subjects stood barefooted with their scapula, buttocks and heels' resting against a wall, the neck was held in a natural non-stretched position and the heels were touching each other. Nutritional status indicators were classified and standardized into Z-scores for height-for-age (HAZ), weight-for-height (WHZ) and weight-for-age (WAZ) in EPI Info (version 3.2), relative to the WHO reference curves recommended for international use WHO [10]. Nutritional assessment of children was evaluated using the World Health Organization [11] recommended HC -for-age specific z-score cut-off value. Moderate under nutrition: <-2 standard deviation (SD) z-score value while severe under nutrition: <-3 standard deviation (SD).

**Data analysis:** the quantitative data was analyzed using Statistical Package for Social Sciences (SPSS) version 15 software package. Data generated were sorted into categories and observations and analyzed by using simple frequency tables and percentages, analysis and Chi-square and at level of significance P< 0.05, were employed to test relationships and differences.

## Results

Table 1 showed that the total prevalence of Intestinal parasitic infections in the study was 16.5%. A total of seven (7) intestinal parasites were reported in the study area. The most prevalent intestinal was *E. histolytica*(5.3%) followed by *A.lumbricoides* (4.0%), while the least prevalent was *Taenia* spp (0.3%). As shown in Table 2, light infection with intestinal helminthes predominated in the study areas, *A. lumbricoides* 75.0%, *Trichuris trichiura* 50.0% and Hookworm 66.7%. Heavy intensity was only recorded in a child infected with *A. lumbricoides* (8.3%) Table 3 shows prevalence of malnutrition in the 300 children used for anthropometric study. The prevalence of stunting (under-height) was 26.0%. Twelve (12) children (4.0%) were under-weight while 4(1.4%) had wasting. As shown in Table 4, the prevalence of severely under- height was higher in non-infected (86.1%) than in infected children (13.9%). Similarly, the prevalence of moderately under-height was higher (83.20%) in non-infected than in infected children (16.7%). The prevalence of moderately underweight was higher in non-infected children (90.0) than in infected children (10.0%), while the prevalence of severely under-weight was similar in both infected and non-infected children (50.0%). The total prevalence of wasting was 1.3%. Majority of the children (infected and non-infected) were well-nourished (98.7%). However, at P - value of 0.587, there was no statistically significant relationship between the prevalence of malnutrition among infected and non-infected children. As shown in Table 5, the total prevalence of anaemia in infected and non-infected children was 17.4%. The prevalence of anaemia was higher (21.1%) in infected children than in non-infected children (16.5). At P = 0.098, there was no significant difference between anaemia in infected and non-infected children. Table 6 shows that out of 232 infected children, 49 (21.1%) had anaemia, 29 (12.5%) mild, 19(8.2%) moderate and 1 (0.4%) severe. Severe anaemia was only reported in children with double infection (2.2%). As shown in Table 7, the least prevalence (41.7%) of malnutrition was recorded among children with light intensity of intestinal helminth infection. Only a child recorded heavy intensity and the child had poor nutrition. However, at (p- value = 0.319) there was no association between prevalence of malnutrition and intestinal helminth infection.

**Table 1:** Total prevalence of intestinal parasite

No Examine	Intestinal parasite	No. Infected	Percentage (%)
300	*lumbricoides*	12	4.0
	*T. trichiura*	2	0.6
	Hookworm	3	1.0
	Taenia spp	1	0.3
	*E. histolytica*	16	5.3
	*G. lamblia*	8	2.7
	*E. coli*	8	2.7
	**Total**	**50**	**16.7**

**Table 2:** Classes of intensity of intestinal helminthes infections level of infection

Class of Intensity (epg)	No. Infected	Percentage (%)
***A. lumbricoides***		
Light (1-4999)	9	75.0
Moderate (5000-49,999)	2	16.7
Heavy (> = 50,000)	1	8.3
**Total**	**12**	**100**
***Trichuris trichiura***		
Light (1-999)	**1**	50.0
Moderate (1000-9,999)	**1**	50.0
Heavy (> = 10, 000)	**0**	0.0
**Total**	**2**	**100**
**Hookworm infection**		
Light (1-999)	2	66.7
Moderate (2000 – 3999)	1	33.3
Heavy (> = 4000)	0	0.0
	**3**	**100**

*Taenia* spp NA = Not available

**Table 3:** Prevalence malnutrition (stunting, under-weight and wasting) in children used for anthropometric

Type of Malnutrition	No Examined	No Malnourished
Height for Age	300	78(26.0%)
Weight for Age	300	12(4.0%)
Weight for Height	300	4(1.4%)
Total	300	94(31.3%)

**Table 4:** Anthropometric Measurement of Infected and Non-infected Children

Class of Malnutrition	No. Examined	Percentage of Malnutrition in Infected	Percentage of Malnutrition in Uninfected
Moderate under-height	42	35(16.7%)	35(83.3)
Severe under-height	36	5(13.9%)	31(86.1)
Moderate under-weight	10	1(10.0%)	9(90.0%)
Severe under-height	2	1(50.0%0	1(50.0%0
Severe wasting	4	0(0.0%0	4(100.0%)
p-value 0.587			

**Table 5:** Prevalence of anaemia in infected and non-infected children

IPI’s Status	Number Examined	Anaemia	Not Anaemic
Infected	232	49(21.1%)	183(78.9%)
Non-Infected	968	160(16.5%)	808(83.5%)
**Total**	**1200**	**209(17.4)**	**991(82.6%)**

X^2^ = 2.7434, df – 1, P- value = 0.098

**Table 6:** Prevalence of anaemia in infected children with respect to number of parasites species

Number of Species	No Examined	Not Anaemic	Mild Anaemia	Moderate Anaemia	Severe Anaemia	Total
Single	188	145 (77.1%)	28(14.9%)	15(8.0%)	0(0.0%)	43
Double	44	38(86.4%)	1(2.3%)	4(9.1%)	1(2.2%)	6
**Total**	**232**	**183(78.9%)**	**29(12.5%)**	**19(8.2%)**	**1(0.4%)**	**49**

P- value = 0.026

**Table 7:** Relationship between varying intensity of helminth infection, malnutrition and anaemia

Class of Intensity for Malnutrition	No. Infected	Malnourished	Not Malnourished
Light	12	5(41.7%)	7(58.3%)
Moderate	4	3(75.0%)	1(25.0%)
Heavy	1	1(100.0%)	0(0.0%)
**Total**	**17**	9	8
**Class of Intensity for Anaemia**		**Anaemic**	**Not Anaemic**
Light	60	2(3.3%)	58(96.7%)
Moderate	21	7(33.3%)	14(66.7%)
Heavy	1	1(100%)	0(0.0%)
Total	**82**	10	72

*Taenia* spp unclassified = 1

P –value = 0.319, for intensity of varying Intensity of Helminth Infection and Malnutrition

P–value = 0.002, for intensity of varying Intensity of Helminth Infection and Anaemia

## Discussion

Many authorities have linked intestinal parasitic infections (mostly helminthes) with an increased risk for nutritional anaemia, protein- energy malnutrition and growth deficits in children, low pregnancy weight gain and intrauterine growth retardation followed by low birth weight [6, 12]. Mechanisms by which intestinal parasitic infections may cause malnutrition exist and these include; impaired nutrient absorption reduction of appetite and resulting infection [4]. In this study, the total prevalence of intestinal parasitic infections was 16.7%, which is low when compared with prevalence of 47.7% reported by Udensi *et al.* [13] in Imo State. In two States in Nigeria, Thomas *et al.* [14] and Orji [15] reported prevalence of 17.75% and 18.0% in Chikun, Kaduna State and Uli community in Anambra State, respectively. The low prevalence reported in this study could be due to the efforts of Imo State Government as the time of the study to reduce child mortality, through improved sanitation, free mass drug administration in different Health Centres, improved personal hygiene through construction of classrooms with modern toilets and sinking of bore-hole water in majority of the schools in urban areas of Imo State. Prevalence of malnutrition (31.3%) observed in the present study was in line with 24% prevalence reported by Amuta *et al.* [16] and 30.0% prevalence reported by Opara *et al*. [6], although, it was far above the observation of WHO [17] that one out of six (16.6%) children in developing countries show signs of malnutrition. Anthropometric values of children showed that most of the children infected with intestinal parasitic infections had normal anthropometric parameters. According to Stephenson *et al.* [18], the impact of intestinal parasitic infections depends on the prevalence rate and intensity of infection. There was relatively low degree of malnutrition and insignificant association with intestinal parasitic infections, underweight and wasting found in few children (4.0% and 1.3% respectively) could be as a result of other factors like poverty and other infections. Intestinal parasitic infections are not the only cause of malnutrition in children, the etiology of malnutrition are multifactorial [19]. The low prevalence of intestinal parasitic infections could explain the reason for insignificant relationship between malnutrition and prevalence of intestinal parasitic infections Total prevalence of stunting was 26.0% and it was not associated with intestinal parasitic infections in the study area. Stunting is mostly due long-term poor nutritional intake and is the best indicator of growth retardation in children over long period of time. The prevalence (16.7%) obtained in the present study was below the prevalence suggested by Stephenson *et al.* [4]. Low prevalence of intestinal parasitic infections may explain the reason for insignificant relationship between intestinal parasitic infections and prevalence of malnutrition. Majority of the children (70.5%) had light infection; hence, it is possible that most of the subjects had acute infection, which is less likely to affect weight and growth in children [20]. Low intensities of intestinal parasitic are known to cause minimal or no clinical impact [20]. Stephenson *et al.* [4] suggested that relationship between helminth infections and nutritional status of young children in a population should be over looked in communities where the prevalence is below 20%. This study suggested that reduction in growth (weight and height) was not associated with intestinal parasitic infection in the study area; it could be due to other health problems and poverty.

The total prevalence of anaemia among infected and non-infected children was 17.4%. Anaemia was insignificantly higher (P=0.098) in infected children than non-infected children. This was in conformity with the study conducted by Orji [15], in Uli, Ihiala Local government Area, Anambra State. The total prevalence of anaemia in infected children was 21.1%. This prevalence was lower when compared with 50% prevalence reported by Ehiaghe *et al.* [21], in infected children in Okada, Edo State, Nigeria. Higher prevalence of anaemia was reported in children that had multiple infections. This was in line with the findings of Ehiaghe *et al.* [21], who reported higher prevalence of anaemia in children infected with multiple intestinal parasites. The higher prevalence observed in children who had double infection could be because of the impact of double morbidity due multiple parasite. Anaemia in infected children varied significantly with respect to location (P = 0.001). With regard to relationship between intensity of intestinal helminth infection and prevalence of anaemia, there was a significant association between varying intensity of helminth infections and prevalence of anaemia in the study area. This finding corroborates with that of Ramdath *et al.* [22], who reported higher prevalence of anaemia in children who were heavily infected with intestinal parasite. On the association between varying intensity of helminth infection and indicators of malnutrition, malnutrition was insignificantly (P=0.319) higher in children who had heavy and moderate intensity of helminth infection than those that recorded light intensity.

## Conclusion

It has been ascertained by some authorities that intestinal parasitic infections can cause increased risk of malnutritional, anaemia, protein- energy malnutrition and growth deficits in children, but in this study, malnutrition and anaemia were not associated with intestinal parasitic infections in this present study. This could be due to light intensity and low prevalence of infection observed in the study areas, as the severity and impact of intestinal parasitic infections depends on the intensity and prevalence rate of infection. The lower prevalence (when compared with other studies in Imo State) recorded in the study areas, has really demonstrated the impact of improved hygiene, provision of potable water and free deworming programmes by the present government and Non-Governmental Organizations on the prevalence of intestinal parasitic infections.

### What is known about this topic

- High intensity of parasitemia had been associated with malnutrition;
- The prevalence of intestinal parasitic infections has been high in developing countries;
- Intestinal parasitic infections has been associated with anaemia.

### What this study adds

- The study adds to baseline epidemiological data on the prevalence and intensity of intestinal parasitic infections in developing countries;
- Knowledge of a disease situation in these the zones studied can be integrated in disease intervention planning;
- Has shown that intestinal parasitic infections were not associated with malnutrition and anaemia, it could be due other factors.

## Competing interests

The authors declare no competing interests.
